# Probiotic has prophylactic effect on spatial memory deficits by modulating gut microbiota characterized by the inhibitory growth of *Escherichia coli*

**DOI:** 10.3389/fnint.2023.1090294

**Published:** 2023-02-21

**Authors:** Jie Zhang, Zengyang He, Lulu Liu, Huailong Li, Tian Wang, Xuefeng Zhu, Yanqing Wang, Dongliang Zhu, Yong Ning, Yi Xu

**Affiliations:** ^1^Anhui Key Laboratory of Tobacco Chemistry, Anhui Tobacco Industrial Co., Ltd., Hefei, China; ^2^School of Food and Bioengineering, Hefei University of Technology, Hefei, China; ^3^Yantai City Center for Disease Control and Prevention, Yantai, China

**Keywords:** probiotic intervention, spatial memory, gut microbiota, *Escherichia coli*, *in utero*

## Abstract

**Background:** The aim of this study is to interrogate the prophylactic effect of probiotic on the lead-induced spatial memory impairment, as well as the underlying mechanisms based on gut microbiota.

**Methods:** Rats were exposed postnatally to 100 ppm of lead acetate during lactation (from postnatal day 1 to 21), to establish the memory deficits model. A probiotic bacterium, namely *Lacticaseibacillus rhamnosus*, was administered by drinking into pregnant rats with a dosage of 10^9^ CFU/rat/day till birth. At postnatal week 8 (PNW8), the rats were subjected to Morris water maze and Y-maze test, with fecal samples collected for 16S rRNA sequencing. Besides, the inhibitory effect of *Lb. rhamnosus* on *Escherichia coli* was carried out in bacterial co-culture.

**Results:** Female rats prenatally exposed to probiotic improved their performances in the behavioral test, indicating that probiotic could protect rats from memory deficits caused by postnatal lead exposure. This bioremediation activity varies depending on the intervention paradigm used. As revealed by microbiome analysis, although administered in a distinct period from lead exposure, *Lb. rhamnosus* further changed the microbial structure disrupted by lead exposure, suggesting an effective transgenerational intervention. Of note, gut microbiota, represented by Bacteroidota, varied greatly depending on the intervention paradigm as well as the developmental stage. The concerted alterations were revealed between some keystone taxa and behavioral abnormality, including lactobacillus and *E. coli*. To this end, an *in vitro* co-culture was created to demonstrate that *Lb. rhamnosus* could inhibit the growth of *E. coli* with direct contact, which is dependent on the growth condition under study. In addition, *in vivo* infection of *E. coli* O157 aggravated memory dysfunction, which could also be rescued by probiotic colonization.

**Conclusions:** Early probiotic intervention could prevent organisms from lead-induced memory decline in later life through reprogramming gut microbiota and inhibiting *E. coli*, providing a promising approach to ameliorate the cognitive damage with environmental origins.

## 1 Introduction

Spatial memory is the ability to remember the layout of the external environment and navigate within its boundaries and normally considered as an important building block of human cognition (Balcerek et al., 2021). As a metal toxicant, developmental lead exposure can damage spatial memory (Xiao et al., 2020), and the impairment usually persists into adulthood. The chelation method was proven ineffective in treating low-level exposures (BLL < 45 μg/dl), and failed to rescue the associated memory deficits (Neal and Guilarte, 2010). Therefore, alternative strategies to attenuate the spatial memory impairment are required to be developed, whereas manipulating gut microbiota has been increasingly appreciated in recent days.

Gut microbiota is a complex community composed of over 1,000 bacterial species, with a majority of taxa categorized as Firmicutes and Bacteroidetes (Liu et al., 2021). Gut microbiota is able to affect brain function through microbiota-gut-brain (MGB) axis, and otherwise, intestinal dysbiosis contributes to a range of neuropathies, including cognition-related psychiatric disorders (Hsiao et al., 2013; Dickerson et al., 2017). As a common approach to adjust gut microbiota, probiotic intake was found potent in improving cognitive function. For instance, long-term probiotic supplementation enhanced memory of middle-aged rats (O’Hagan et al., 2017); *Lactobacillus helveticus* NS8 attenuated the cognition deficits caused by restraint stress (Liang et al., 2015). In addition, our previous findings also indicated the positive influence of probiotics on lead-induced memory dysfunction (Xiao et al., 2020; Gu et al., 2022), but till date, no attempts have been made to interrogate their preventive roles prior to the actual damage caused by environmental insults.

Like some probiotic strains, *Lacticaseibacillus rhamnosus* (formerly known as *Lactobacillus rhamnosus*) participates in bioremediation, which is defined as a process for lactobacilli to bind to and remove certain heavy metals and other toxins (Petrova et al., 2021). The absorbent properties enable *Lb. rhamnosus* to immobilize lead and cadmium as a cell-surface cluster (Daisley et al., 2019). This finding might propose *Lb. rhamnosus* as a competent candidate to offer protection for lead-induced memory deficits. Of note, an open-label study showed that, when yogurt containing lactobacillus strains was continuously given to pregnant women in a rural Tanzanian population, the gut microbiota of the newborn was profoundly reprogrammed while mothers’ microbiota remained constant (Bisanz et al., 2015). This means that offspring microbiota is susceptible to maternal probiotic intake, which is thus hypothesized to impose benefits on CNS. But still, the mystery is required to be resolved as to whether the transgenerational intervention can take place with regards to developmental memory injury.

Therefore, a gestational exposure paradigm was invented here to study the influence of *Lb. rhamnosus* on spatial memory, with paradigm/duration specificity, microbiota changes as well as implication of *E. coli* investigated in sequence. This study is an intriguing example of *in utero* use of probiotic to ameliorate memory decline in later life, shedding light on the microbe-based nutritional prevention of neurological disorders.

## 2 Materials and methods

### 2.1 Animals and study design

Sprague-Dawley (SD) rats were obtained from the Laboratory Animal Center of Anhui Medical University. All animal manipulations were approved by the Institutional Animal Care and Use Committee of Hefei University of Technology, China. Three dams were used in each treatment group, and each dam could give birth to 8–11 offspring on average. The pups were randomly selected after weaning and subjected to subsequent trials. The lead acetate (125 ppm) was administered *ad libitum* in drinking water during lactation, that is, from PND1 to PND21. *Lacticaseibacillus rhamnosus* GR-1 (LGR-1) was collected from the mid-exponential stage (OD_600 nm_ = 1.0) and supplied at a dosage of 10^9^ organisms/rat/day in sterilized water during pregnancy (from parental cohousing to birth) in a tailored container, which bears a long neck for the cagemates accessible to drink, and the drinking was routinely completed in 2 min. This administration should also be seen in the supplementary video in our previous study (Xiao et al., 2020). Besides, intervention and repair modes were performed according to the same paradigm, except in a separate duration of lactation (PND1–21) and postweaning (PND22 till sacrifice after behavioral test at PNW8), respectively. Female rats were subjected to the subsequent experiments due that no uniform memory damages were observed for male SD rats under the tested condition (Xiao et al., 2020).

### 2.2 Bacterial strains and growth conditions

*Lacticaseibacillus rhamnosus* GR-1 was grown at 37°C in de Man, Rogosa and Sharpe (MRS) medium (Oxiod, Basingstoke, England). *E. coli* O157:H7 was grown at 37°C under aeration in Luria-Bertani (LB) medium. Their *in vivo* use was performed when new culture reached OD_600 nm_ = 1.0 and bacteria were harvested by centrifugation. The viable bacterial number was calculated through plate counting with serial dilutions. The *E. coli* infection was carried out by supplying animals with 10^9^ organisms/rat/d in sterilized water in a tailored container from weaning till PNW8, in the presence or absence of lead. The probiotics were given at the same developmental stage as *E. coli* infection, to study the mitigating effect.

Co-culture experiments were carried out using MRS-LB medium, which was formulated by mixing MRS and LB medium with a ratio of 1:1. The pH of the medium was adjusted with NaOH and HCl. The volume of culture was set as 10 ml, and 100 μl (1%) of O157 strain was first inoculated, followed by LGR-1 with varying dosages, incubation time, and pH values. The co-culture was incubated at 37°C without aeration till collection for enumeration. O157 was counted using an LB medium and identified through distinct colony morphology. Survival rate was calculated as the ratio of viable cell number of each treatment to the untreated group. In addition, supernatant of LGR-1 was collected at the mid-exponential stage and added to *E. coli* culture for 24 h with varying dosages, and OD_600 nm_ value was measured through the Microplate Reader (Thermo Scientific, Beijing, China).

Propidium iodide (PI) staining was used here to detect bacterial membrane damage. Following co-culture growth, 1 ml bacteria were centrifuged at 5,000 *g* for 3 min and washed twice with sterile PBS. The harvested cells were then incubated with PI (3 μl per ml) in the dark at 37°C for 15–20 min. After washing twice with PBS to remove excess dye, the suspension was then transferred to a glass slide for fluorescence microscopy examination (ECLIPSE Ti2-U, Nikon, Tokyo, Japan), and the relative fluorescence was calculated from five respective images using Image J (Bethesda, MD, USA).

### 2.3 Behavioral test to evaluate spatial memory

A behavioral test was conducted as previously described (Xiao et al., 2020) with some modifications: the assessment began at PNW8, and all animal traces were video-recorded and automatically scored by Smart tracking software (ANY-maze; Stoelting, Shanghai, China). The whole blood samples were gathered from the femoral vein. And after sample collection at PNW10, blood lead levels were determined using the double channel atomic fluorescence spectrometer (TITAN Instruments, Beijing, China) following nitric acid treatment, according to the standard protocol.

The Morris Water Maze (MWM) test was performed in a circular pool with a diameter of 1,600 mm and depth of 700 mm, which was filled with water to a depth of 400 mm. The temperature was maintained at 25°C during test. After 5 days of training, the platform was removed on the test day and each rat was given a 90 s chance to locate the hidden platform, which was used to reflect its ability of memory retention. Using ANY-maze, latency to the first entry, crossing times and duration spent on the target quadrant, as well as total moving distances were analyzed.

The Y-maze test was performed as follows: the rat was placed in the central platform and then allowed to explore all three arms of the maze. The number of spontaneous alternations (defined as the number of successive triplet entries into three arms without any repeated entries) was monitored in a 10 min test session, and such an order of three entries should be regarded as one time of alteration. The percentage of spontaneous alternation was calculated as the ratio of “alternation number” with “total entries—2”.

### 2.4 16S rRNA sequencing and data analysis

16S rRNA sequencing was used to unveil intestinal microbiome composition under varying conditions. The fresh feces from female rats were collected at PND68, homogenized, and subjected to DNA extraction. Subsequently, 1 ng/μl of DNA was subjected to 16SV4 rRNA amplification. The PCR products were purified and subjected to IonS5TMXL (ThermoFisher, Beijing, China) for sequencing. Data analysis was conducted with the following steps: data split, data filtration, chimera removal, OTU production, species annotation, phylogenetic construction, and data normalization.

Differences in microbial communities between groups were analyzed by phylogeny-based unweighted UniFrac distance metrics. Alpha diversity and UPGMA clustering were performed with the respective QIIME scripts (Version 1.9.1). R software (Version 4.0.3) was used to analyze the inter-group differences of beta diversity and graphs were invented through the ggplot2 package. LEfSe analysis was performed using LEfSe software according to the manufacturer’s instructions, with the default filter value of the LDA score set as 4.0. The sequencing data was then submitted to the NCBI database, and got an accession number of PRJNA906312.

### 2.5 Statistical analysis

Graph data were presented as means ± SEM. Statistical analysis was performed using SPSS software. One-way ANOVA was used to perform inter-group analysis with multiple treatments, and an unpaired, two-tailed t-test was used to perform two group comparisons where necessary. Two-way ANOVA was performed to calculate the interaction effect of Pb exposure and O157 infection. PCA analysis was performed and scaled to unit variance (R function prcomp). Permanova/adonis was used to compare the complexity of different microbial communities. Correlation analysis between *E. coli* abundance and the ratio of distances traveled in the target quadrant was performed by the software Graphpad prism 8, with only the fecal samples obtained at PNW8 from prevention- and repair-treated rats as well as the controlled and lead-exposed rats considered. The number of samples examined in each analysis was shown in the respective figure legends.

## 3 Result

### 3.1 Maternal supplementation of probiotic strain prevents the offspring’s memory deficits

**Figure 1:**
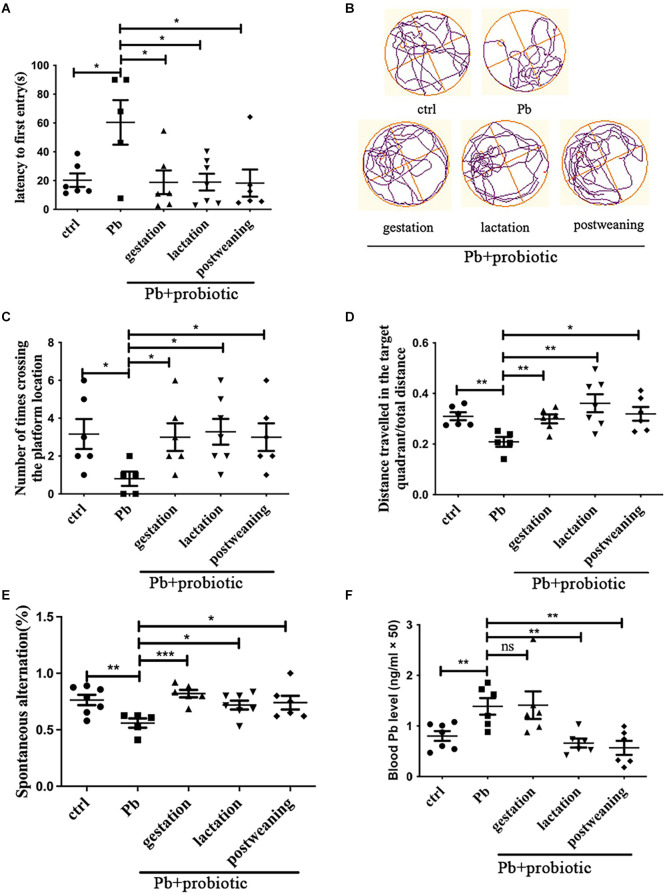
Behavioral assessment of rats in response to Pb and probiotic treatment. Panels **(A–D)** represent Morris water maze analysis (*n* = 5–7), which are calculated with respect to latency to the first entry **(A)**, number of crossing times **(C)** and distance traveled in the target quadrant **(D)**. The representative moving tracks of rats on the test day were shown in **(B)**. Panel **(E)** represents the spontaneous alteration obtained from the Y-maze test (*n* = 5–7). Panel **(F)** represents the blood lead levels in the various treatment groups (*n* = 6–7). Statistical analysis was performed using one-way ANOVA or unpaired *t-*test. All data are expressed as mean ± SEM. ns, *P* > 0.05; *P < 0.05; **P < 0.01; ***P < 0.001. ctrl, untreated rats; Pb, lead-exposed rats during lactation; probiotic, *Lacticaseibacillus rhamnosus*. Gestation, lactation, and postweaning represent the different treatment periods of *Lb. rhamnosus*, respectively.

Developmental lead exposure is a widely-accepted approach to establish the spatial memory deficit model (Soleimani et al., 2016). In this study, 125 ppm of lead acetate was exposed to female SD rats during lactation (PND1–21), and memory deficits were detected at PNW8, as evidenced by the Morris water maze and Y-maze test (Figures 1A–E). When LGR-1 was *in utero* administered (given from parental cohousing till birth), rats displayed significantly better performance in the water maze test, manifested by latency to the first entry (Figure 1A), number of entries (Figure 1C), and distance traveled in the target quadrant (Figure 1D), indicating an improved spatial memory compared to lead-induced adversity. The representative moving tracks were recorded and shown in Figure 1B. During the test, no locomotion variations were observed among the groups (Supplementary Figure S1). Besides, the prophylactic effect of probiotic strain was also validated in a Y-maze test (Figure 1E).

The intervention paradigms during lactation (LGR-1 given from PND1 to PND21) and postweaning (LGR-1 given from PND22 to sacrifice after behavioral test starting at PNW8) to were also carried out to fully decipher the CNS-modulating activity of probiotic strain. According to the results (Figures 1A–E), just like gestational intervention, both lactational, and postweaning intervention could rescue the memory deficits. As for working memory presented by the Y-maze test, it was found that lead exposure also significantly decreased the alteration frequency of the tested rat (*P* < 0.01), which was reversed, to a variable extent, by the probiotic supplement in the gestational, lactational, and postweaning period, respectively. Among them, the gestational intervention produced the most prominent mitigation, as demonstrated by the significance of differences (*P* < 0.001). Nonetheless, when blood lead levels were measured, it was found that both treatments reduced lead concentration, in contrast with cases in prophylactic group (Figure 1F). This result might indicate that maternal supplementation of probiotics mitigates spatial memory independent of lead bioremediation.

### 3.2 Probiotic strain exerts prophylactic effect and simultaneously reshapes gut microbiota

**Figure 2:**
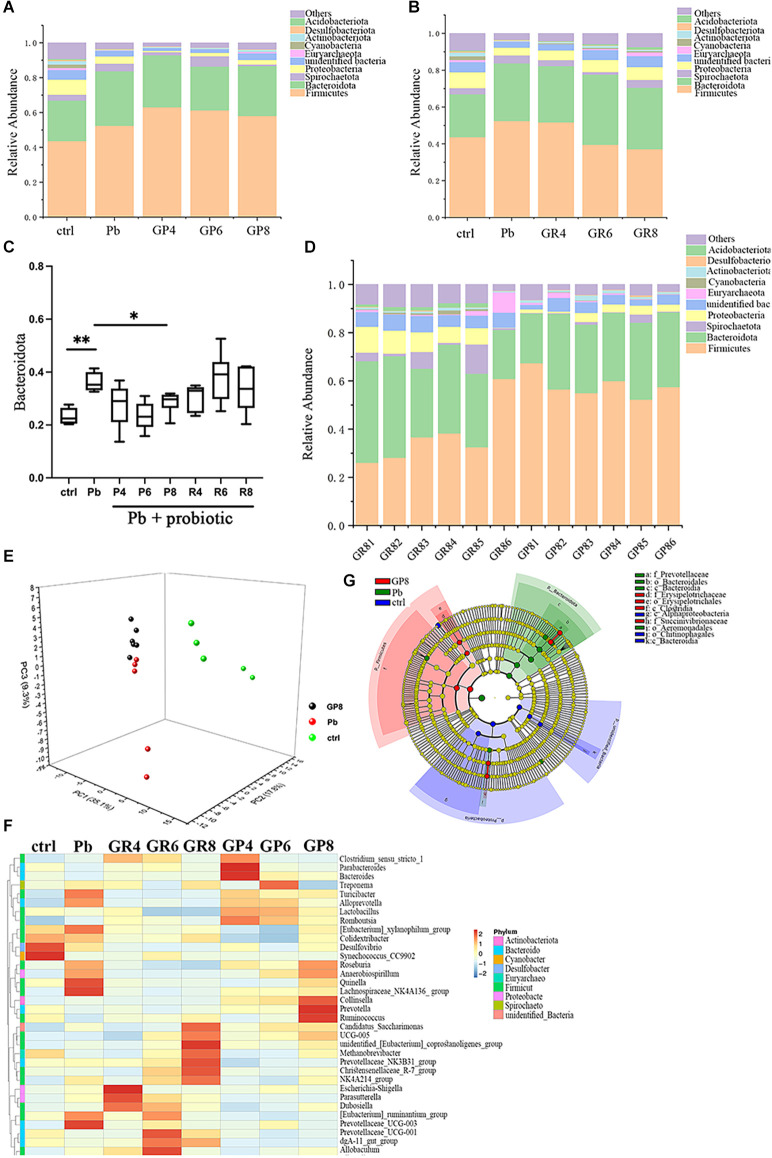
Gut microbiota analysis in response to Pb and probiotic treatment (*n* = 5–6). Panels **(A,B,D)** represent stacked bar charts showing microbiota composition at phylum level. ctrl, untreated rats; Pb, lead-exposed rats during lactation; G, LGR-1; P, prevention mode indicating gestational supplementation of probiotic; R, repair mode indicating postweaning supplementation of probiotic. The following number indicates the # of the postnatal week. And the combination of letters and number signify their integral meanings. ctrl and Pb were sampled at PNW8. Panel **(C)** represents the relative abundance of Bacteroidota in various groups. Panel **(E)** represents PCA analysis of the gut microbiota upon each treatment. Panel **(F)** represents the heatmap of representative tax. **(G)** Microbial taxa identified using LEfSe analysis (LDA score > 4.0, *P* < 0.05) across the tested groups (ctrl, Pb, Pb+probiotic-P8). Statistical analysis was performed using one-way ANOVA corrected by Tukey with respect to Bacteroidota abundance; Permanova/Adonis analysis was used to compare different groups in PCA analysis. All data are expressed as mean ± SEM. *P < 0.05; **P < 0.01.

In order to explore the precise route that *Lb. rhamnosus* improves spatial memory, the offspring gut microbiota was subjected to 16S rRNA sequencing (Figures 2A–G, Supplementary Figures S2A–E). In Figure 2A, “ctrl” and “Pb” represent the untreated and lead-exposed rats during lactation respectively, as indicated above, while GP represents the prevention mode of LGR-1 intervention as shown above, and fecal samples were collected and analyzed at the postnatal 4, 6 and 8 weeks respectively. And the following panels abide by similar designation rules. As unveiled by the overall structure, the abundance of Bacteroidetes was increased by lead exposure, which was further reversed by prenatal probiotic supplement (Figures 2A–C). This intervention is dependent on the treatment period, as this rescue is only significant at PNW8 instead of PNW4 and 6. In addition, postweaning treatment led to a distinct microbial composition, with changes in Firmicutes highly implicated (Figure 2B). When gut microbiota from different modes were compared, samples from PNW8 showed robust discrepancy with regards to overall phylum composition, as represented by the apparent changes in Firmicutes abundance (Figure 2D). The data suggested that the window of probiotic treatment might result in divergent microbiome composition, emphasizing the specificity of preventive effect of LGR-1. Besides, despite the overall differences between repair and prevention modes, there still exists apparent individual variability for gut microbiome, as manifested by GR1–6. Taken together, this data suggests that probiotics could influence gut microbiota in a unique transgenerational way.

In a PCA analysis, lead exposure reshaped gut microbiota and probiotic further modified this tendency at PNW8, exhibiting the proximal coordinates with untreated rats (Figure 2E). This graph gave the visual evidence that feeding rats with *Lb. rhamnosus* prenatally improves the lead-led dysbiosis. In terms of representative taxa, it was revealed that some highly-abundant bacteria, exemplified by Prevotellaceae and *Escherichia-Shigella* (Figure 2F), was seemingly in parallel with Pb/probiotic treatment as well as the consequent memory alteration. Their involvement was further substantiated in the LEfSe analysis, wherein roles of Prevotellaceae (Figure 2G) were further underpinned. Specifically, Erysipelotrichaceae, Erysipelotrichales, Clostridia, and Succinivibrionaceae were enriched in the LGR-1 intervention group, while Prevotellaceae, Bacteroidales, Bacteroidia, and Aeromonadales were highlighted in the Pb-exposed rat intestines (LDA > 4, *P* < 0.05). Besides, the taxa with top 10 abundance were listed in Tables s 1–3, as classified by family, genus, and species respectively.

**Table 1:** The top 10 family of microbial taxa as identified by 16S sequencing.

**Taxonomy**	**ctrl**	**Pb**	**GR4**	**GR6**	**GR8**	**GP4**	**GP6**	**GP8**
Lactobacillaceae	0.253808 ± 0.0869	0.232866 ± 0.0415	0.281908 ± 0.1073	0.106257 ± 0.0334	0.112668 ± 0.0453	0.42175 ± 0.124	0.407792 ± 0.1666	0.292631 ± 0.0797
Prevotellaceae	0.093283 ± 0.0322	0.17326 ± 0.0561	0.09913 ± 0.0529	0.136517 ± 0.0585	0.166752 ± 0.0874	0.096915 ± 0.0575	0.116887 ± 0.0588	0.155401 ± 0.0453
Spirochaetaceae	0.032911 ± 0.0335	0.04311 ± 0.0291	0.031932 ± 0.0349	0.014101 ± 0.0083	0.041732 ± 0.0456	0.018081 ± 0.0311	0.059001 ± 0.0860	0.009206 ± 0.0124
Lachnospiraceae	0.081657 ± 0.0132	0.1135 ± 0.0459	0.071439 ± 0.0156	0.054623 ± 0.0156	0.055949 ± 0.019	0.075439 ± 0.0269	0.073161 ± 0.0287	0.118504 ± 0.0453
Muribaculaceae	0.070452 ± 0.0159	0.084676 ± 0.0291	0.136594 ± 0.0234	0.149386 ± 0.0106	0.084381 ± 0.0178	0.094717 ± 0.0391	0.069154 ± 0.017	0.079413 ± 0.0176
Erysipelotrichaceae	0.011417 ± 0.0034	0.030097 ± 0.0160	0.056893 ± 0.0466	0.06655 ± 0.0636	0.021613 ± 0.0142	0.014121 ± 0.0094	0.028355 ± 0.0098	0.032323 ± 0.0223
Bacteroidaceae	0.027216 ± 0.0084	0.02036 ± 0.0072	0.029294 ± 0.009	0.026165 ± 0.0129	0.026653 ± 0.0097	0.075897 ± 0.0283	0.04038 ± 0.0353	0.031627 ± 0.0113
Rikenellaceae	0.010771 ± 0.0051	0.019885 ± 0.007	0.017805 ± 0.008	0.040374 ± 0.0275	0.026316 ± 0.0125	0.015589 ± 0.0069	0.012946 ± 0.0059	0.010684 ± 0.0049
Methanobacteriaceae	0.012513 ± 0.0109	0.00155 ± 0.002	0.000228 ± 0.0001	0.007433 ± 0.0108	0.020475 ± 0.0318	0.000144 ± 0.0006	0.000379 ± 0.0008	0.00796 ± 0.0077
Succinivibrionaceae	0.001485 ± 0.0011	0.022845 ± 0.0343	0.002929 ± 0.0013	0.002681 ± 0.0011	0.003069 ± 0.0026	0.004846 ± 0.0034	0.015641 ± 0.0128	0.023823 ± 0.0126

R, repair mode; P, prevention mode; the number indicates the # of the postnatal week.

**Table 2:** The top 10 genus of microbial taxa as identified by 16S sequencing.

**Taxonomy**	**ctrl**	**Pb**	**GR4**	**GR6**	**GR8**	**GP4**	**GP6**	**GP8**
*Lactobacillus*	0.2537 ± 0.0870	0.2328 ± 0.0416	0.2819 ± 0.1073	0.1062 ± 0.0334	0.1126 ± 0.0454	0.4216 ± 0.1241	0.4077 ± 0.1666	0.292617 ± 0.0798
*Treponema*	0.0211 ± 0.026	0.0333 ± 0.0197	0.0303 ± 0.0351	0.0127 ± 0.0081	0.0375 ± 0.0428	0.0171 ± 0.0314	0.0571 ± 0.0848	0.0053 ± 0.0054
Prevotellaceae_NK3B31_group	0.0416 ± 0.0259	0.0475 ± 0.0249	0.0442 ± 0.0352	0.0617 ± 0.0324	0.1119 ± 0.0725	0.0111 ± 0.0050	0.0238 ± 0.0265	0.0255 ± 0.0132
*Prevotella*	0.0328 ± 0.0176	0.0160 ± 0.0026	0.0225 ± 0.0127	0.0131 ± 0.0049	0.0213 ± 0.0100	0.0343 ± 0.0163	0.0305 ± 0.0195	0.0947 ± 0.0477
*Bacteroides*	0.0272 ± 0.0085	0.0204 ± 0.0073	0.0293 ± 0.0091	0.0262 ± 0.0130	0.0267 ± 0.0098	0.0759 ± 0.0284	0.0404 ± 0.0354	0.0316 ± 0.0113
*Allobaculum*	0.0049 ± 0.0021	0.0119 ± 0.0113	0.0227 ± 0.0197	0.0408 ± 0.0454	0.0094 ± 0.0063	0.0040 ± 0.0018	0.0087 ± 0.0037	0.0119 ± 0.0063
*Alloprevotella*	0.0032 ± 0.0016	0.0424 ± 0.0178	0.0096 ± 0.0033	0.0124 ± 0.0085	0.0083 ± 0.0055	0.0354 ± 0.0307	0.0342 ± 0.0390	0.0172 ± 0.0073
Lachnospiraceae_NK4A136_group	0.0075 ± 0.0049	0.0333 ± 0.0349	0.0098 ± 0.0075	0.0076 ± 0.0026	0.0090 ± 0.0070	0.0156 ± 0.0085	0.0141 ± 0.0144	0.0184 ± 0.0114
Prevotellaceae_UCG-003	0.0051 ± 0.0043	0.0555 ± 0.0274	0.0113 ± 0.0042	0.0275 ± 0.0154	0.0105 ± 0.0026	0.0090 ± 0.0057	0.0222 ± 0.0139	0.0076 ± 0.0045
*Methanobrevibacter*	0.0125 ± 0.0110	0.0016 ± 0.0021	0.0002 ± 0.0002	0.0074 ± 0.0108	0.0205 ± 0.0318	0.0001 ± 0.0001	0.0004 ± 0.0008	0.0080 ± 0.0078
*Escherichia-Shigella*	0.0004 ± 0.0006	0.0021 ± 0.0004	0.0138 ± 0.0137	0.0006 ± 0.0005	0.0006 ± 0.0007	0.0005 ± 0.0003	0.0024 ± 0.003	0.0004 ± 0.0004

R, repair mode; P, prevention mode; the number indicates the # of the postnatal week.

**Table 3:** The top 10 species of microbial taxa as identified by 16S sequencing.

**Taxonomy (Species)**	**ctrl**	**Pb**	**GR4**	**GR6**	**GR8**	**GP4**	**GP6**	**GP8**
*Lactobacillus murinus*	0.0825 ±0.0774	0.1043 ±0.0262	0.1247 ± 0.0934	0.0374 ± 0.0176	0.0421 ± 0.0172	0.0473 ± 0.0136	0.0567 ± 0.0252	0.0556 ± 0.0353
*Lactobacillus reuteri*	0.0614 ± 0.0334	0.0344 ± 0.0141	0.0332 ± 0.0148	0.0250 ± 0.0126	0.0226 ± 0.0099	0.0499 ± 0.0076	0.0432 ± 0.0221	0.0261 ± 0.0067
*Trichinella pseudospiralis*	0.0003 ± 0.0003	0.0143 ± 0.0260	0.0012 ± 0.0015	0.0014 ± 0.0007	0.0026 ± 0.0011	0.0015 ± 0.0027	0	0.0010 ± 0.0021
*Prevotella copri*	0	0.0016 ± 0.002	0.0004 ± 0.0008	0.0005 ± 0.0013	0.0001 ± 0.0003	0.0143 ± 0.0123	0.0137 ± 0.0119	0.0332 ± 0.0194
*Bacteroides caecigallinarum*	0	0.0007 ± 0.0011	0	0.0003 ± 0.0007	0	0.0088 ± 0.0072	0.0130 ± 0.0225	0.0030 ± 0.0014
*Romboutsia ilealis*	0.0058 ± 0.0027	0.0096 ± 0.0019	0.0141 ± 0.0043	0.0121 ± 0.0025	0.0099 ± 0.0046	0.0243 ± 0.0108	0.0205 ± 0.0099	0.0148 ± 0.0089
*Bacteroides sartorii*	0.008 ± 0.0065	0.003 ± 0.0025	0.0055 ± 0.0046	0.0016 ± 0.0013	0.0025 ± 0.0021	0.0179 ± 0.0144	0.0036 ± 0.0022	0.0046 ± 0.0033
*Escherichia coli*	0.0004 ± 0.0006	0.0021 ± 0.0004	0.0138 ± 0.0137	0.0006 ± 0.0005	0.0006 ± 0.0007	0.0005 ± 0.0003	0.0025 ± 0.003	0.0005 ± 0.0004
*Helicobacter apodemus*	0.0014 ± 0.0007	0.0007 ± 0.0005	0.0015 ± 0.0007	0.0037 ± 0.006	0.0022 ± 0.0014	0.0004 ± 0.0003	0.0021 ± 0.0046	0.0005 ± 0.0002
*Faecalibaculum rodentium*	0.0000	0.0021 ± 0.0029	0.0012 ± 0.0026	0.0001 ± 0.0001	0.0002 ± 0.0002	0.0008 ± 0.0009	0.0046 ± 0.0057	0.0007 ± 0.0006

R, repair mode; P, prevention mode; the number indicates the # of the postnatal week.

In summary, *Lb. rhamnosu*s exerts prophylactic effect on spatial memory and simultaneously reshapes offspring gut microbiota.

### 3.3 *Lb. rhamnosus* inhibits the growth of *E. coli* both *in vivo* and *in vitro*

**Figure 3:**
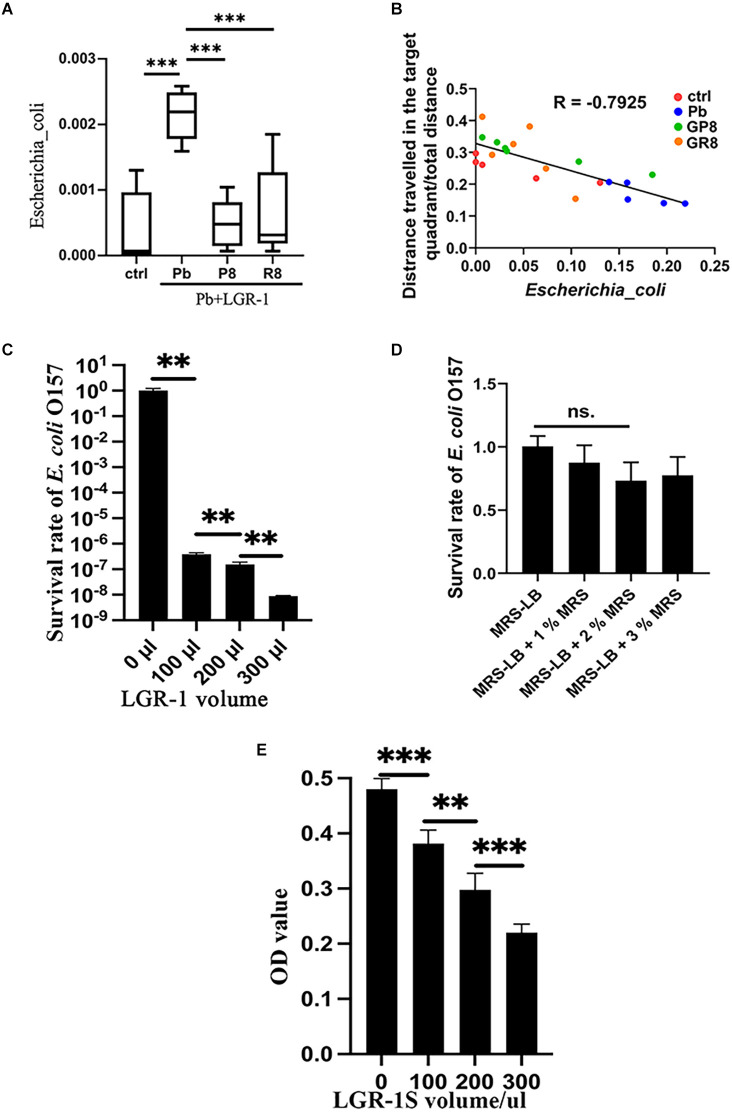
Inhibitory effect of *Lb. rhamnosus* on *E. coli* *in vivo* and *in vitro*. **(A,B)** The relative abundance of *E. coli* **(A)** across groups, as revealed by 16S rRNA sequencing (*n* = 5–6). **(B)** Correlation analysis between the relative abundance of *E. coli* and memory deficits. The memory deficits were expressed as the percentage of distances covered in the target quadrant; The values of *E. coli* abundance were here amplified 100 times to make it better legible in the graph. The data is summarized from the respective groups with variable colors: ctrl, Pb, Pb+LGR-1 (P8), and Pb+LGR-1 (R8); Panels **(C–E)** represent the co-culture experiment of *Lb. rhamnosus* and *E. coli* (*n* = 4). The viable cells of *E. coli* O157 were calculated with plate counting **(C)** when a varying volume of LGR-1 was provided to 100 μl of *E. coli* and incubated in a mixed medium for 24 h. The influence of the additional MRS medium on the survival of *E. coli* was studied and compared as a controlled condition **(D)**. The supernatant of LGR-1 (LGR-1S) was tested on its ability to inhibit *E. coli* **(E)**. ctrl, untreated rats; Pb, lead-exposed rats during lactation; G, LGR-1; P, prevention mode indicating gestational supplementation of probiotic; R, repair mode indicating postweaning supplementation of probiotic. The following number indicates the # of the postnatal week. And the combination of letters and number signify their integral meanings. Statistical analysis was performed using one-way ANOVA with Tukey correction for multiple comparisons or unpaired *t-*test. All data are expressed as mean ± SEM. ns, *P* > 0.05; **P < 0.01; ***P < 0.001. LGR-1, *Lacticaseibacillus rhamnosus* GR-1.

Among the key taxa, *E. coli* receives much attention due to its prevalence and pathogenic property (Iwasaki et al., 2022). By analyzing the abundance of *E. coli* in rat intestines, this species was found to be stimulated by lead exposure and returned to normal when probiotic intake was performed and samples were collected at PNW8 (Figure 3A). This trend did not vary depending on the treatment paradigm, as both prenatal and postweaning interventions imposed similar outcomes. Furthermore, as revealed by correlation analysis (Figure 3B), *E. coli* is closely related to the host performance in behavioral trial with a correlation coefficient of −0.7925 (*P* < 0.001), indicating it has an impact on spatial memory.

We then investigated the abundance of lactobacillus in various conditions. As shown in Supplementary Figure S2E, the abundance of lactobacillus did not comply with a consistent pattern, except for displaying inverse propensity with *E. coli* in the context of changing trajectory presented by Pb- and P4-treated group. To further study the influence of *Lb. rhamnosus* on *E. coli* O157:H7, we conducted co-culture experiments *in vitro*. By monitoring the growth curve of *E. coli* in response to LGR-1 invasion, a strong inhibitory effect was detected when probiotic was added in a ratio of 3:1 and growth lasted up to 24 h (Figure 3C, Supplementary Figures S3A–C). The decreased survival of *E. coli* is unlikely due to the influence of the added volume of MRS medium, as demonstrated in Figure 3D. Besides, some variations of cell survival were observed under different pH values (Supplementary Figures S3D–G). During the process, membrane integrity was damaged as evidenced by PI staining and fluorescence intensity (Supplementary Figures S4A,B). Subsequently, the supernatant of LGR-1 culture was found potent in controlling the growth of *E. coli* (Figure 3E), suggesting that secretory substances play essential roles.

Overall, *E. coli* is inhibited by *Lb. rhamnosus*, which may be associated with memory protection.

### 3.4 *Lb. rhamnosus* improves spatial memory aggravated by *E. coli*

**Figure 4:**
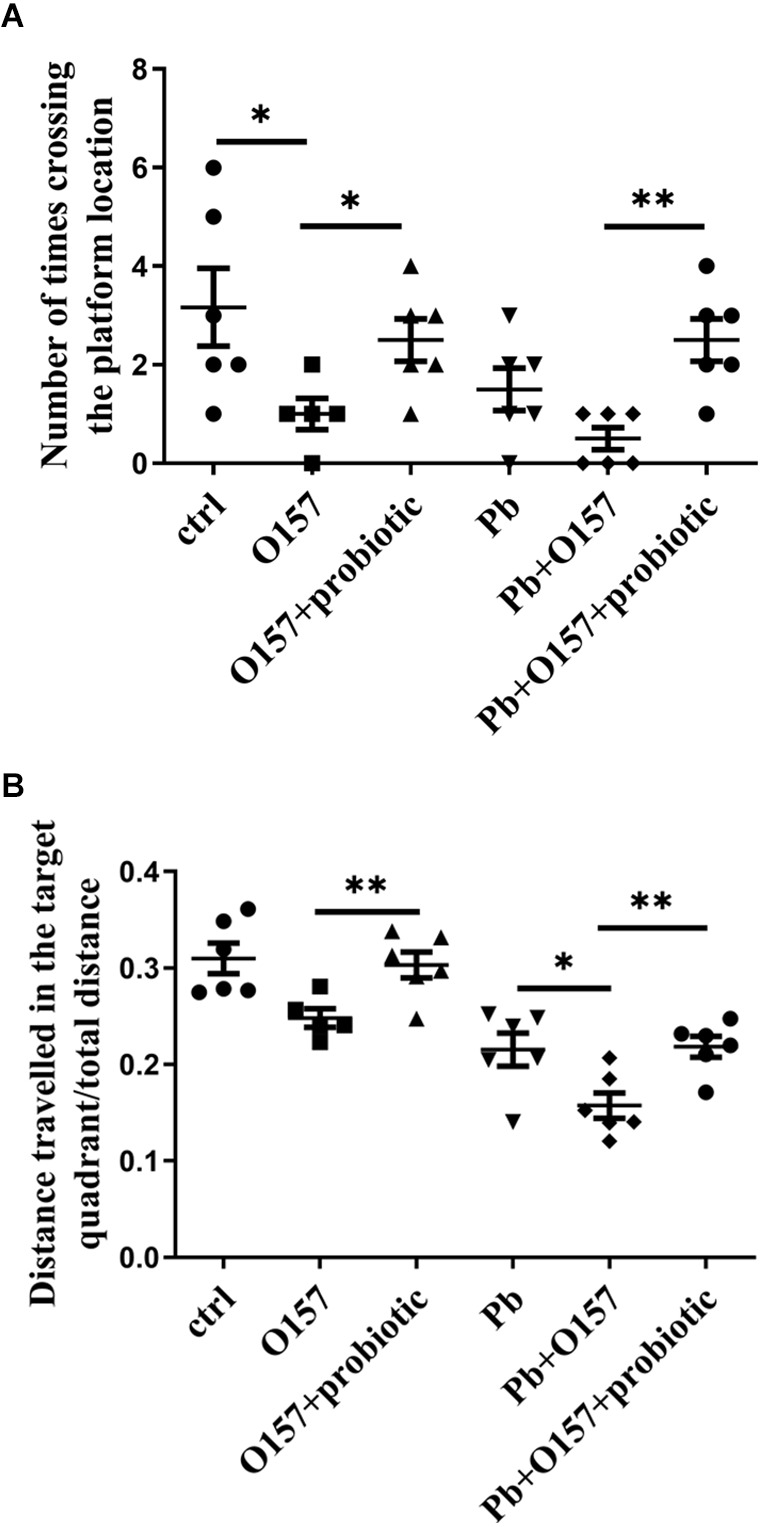
Morris water maze test of rats in response to O157 (or O157+Pb) and probiotic treatment. The number of crossing times **(A)** and distance traveled in the target quadrant **(B)** in response to O157, Pb, and probiotic treatment. Both infection of *E. coli* (10^9^/rat/d) and the addition of *Lb. rhamnosus* were performed from weaning till PNW6, and memory status was tested at PNW8. ctrl, untreated rats; Pb, lead-exposed rats during lactation, O157 applied from weaning till PNW8, probiotic, LGR-1 applied from weaning till PNW8, and “+” means all the involved manipulations were conducted. Statistical analysis was performed using one-way ANOVA, two-way ANOVA or unpaired *t-*test. All data are expressed as mean ± SEM. *P < 0.05; **P < 0.01.

We then investigate if probiotic offers protection from memory decline caused or aggravated by *E. coli* infection. To this end, O157 was fed into rats with or without lead exposure from weaning to PNW8. According to the water maze test, the infection of this pathogen aggravated memory deficits, or alternatively led to a comparable situation with lactational lead exposure (Figures 4A,B). This result indicates that the *E. coli* strain contributes to the severity of memory deficits. Next, probiotic was supplemented to rats at the same developmental stage as O157. It was then seen from behavioral assessment, that probiotic strain rescued memory decline mediated by O157 (Figures 4A,B). Moreover, probiotic and O157 infections showed interaction in causing deficits of distance traveled in the target quadrant in the absence (*P* = 0.0086) and presence (*P* = 0.0255) of Pb exposure, as analyzed by two-way ANOVA with correction of Geisser-Greenhouse. In line with the inhibitory effect on *E. coli*, it could deduce that *Lb. rhamnosus* antagonizes the memory deficits influenced by *E. coli* infection.

## 4 Discussion

In this study, a new prophylactic function of *Lb. rhamnosus* was discovered to associate with lead-induced spatial memory deficits. It is noteworthy that the prevention mode did not allow for the direct contact of this probiotic strain with lead acetate, which was achieved by the temporal separation of the treatment paradigm. Under this tested occasion, probiotics did not cause a reduced blood lead level as expected, but still effectively improved memory impairment. This finding might have relevance with the gut microbiota-reshaping and *E. coli*-inhibitory activity of *Lb. rhamnosus*, showing a unique intervention of lead neurotoxicity.

Lead/Pb is a ubiquitous environmental toxicant, with adverse psychiatric consequences including memory deficits (Schneider et al., 2013). To ameliorate lead toxicity, chelation therapy as well as bioremediation of lactobacillus were used to reduce blood lead levels. However, this therapy did not merit coping with low-level exposures (BLL < 45 μg/dl; Daisley et al., 2019), and failed to reverse the related memory deficits (Neal and Guilarte, 2010). Therefore, an alternative strategy is warranted to be developed to protect organisms from memory decline caused by long-term chronic lead exposure. Our previous finding demonstrated the feasibility of adjusting gut microbiota by probiotic intake (Xiao et al., 2020), however, the preventive validity of single bacterium was not well studied. The probiotic activity shown here is consistent with a prior report that *Lb. rhamnosus* could immobilize lead* in vitro* (Daisley et al., 2019), as both lactational and postweaning treatment decreased lead concentration (Figure 1F). Still, as revealed by *in utero* administration, there must exist a distinct route to mediate LGR-1’s microbiota-modulating activity, as well as the ensuing memory improvement. Still, it could not be excluded that the overall health status of rats was improved by gestational probiotic intake instead of a specifying brain-moderating action, which might request further clarification.

Intricate relations exist between the intestinal bacterial community and CNS. For instance, injection of *Lactobacillus rhamnosus* JB-1 improved the emotional behavior of the mouse (Bravo et al., 2011) by regulating MGB axis. In our previous study (Xiao et al., 2020), the dysbiosis induced by Pb was corrected, which caused memory repair, as demonstrated by fecal microbiota transplant. Therefore, by optimizing the microbial community, LGR-1 is able to influence the memory status as shown in this study. Given that the unique MGB axis mediates the specific gut-brain crosstalk, our efforts are made to delineate the mediatory route in probiotic effect, manifested by modulating the immune-microglia route (Gu et al., 2022). Another valuable reference is the intervention process of formulated probiotics (Xiao et al., 2020), which showed that IL-6/H3K27me3 played important roles in improving spatial memory. Different from these reports, the current study mainly concentrates on the transgenerational probiotic effect, which should be regarded as the preceding event of MGB functioning.

It is found here maternal supplementation of *Lb. rhamnosus* could change the offspring’s microbiota. As the transgenerational intervention was first discovered with respect to this probiotic bacterium, this result is not difficult to elaborate due to the predominant contribution of maternal GM to the infant microbiota (Ferretti et al., 2018). Microbes from the maternal intestine normally persist in the infant habitat, and gradually prevail over niche competitors, which might constitute the major force driving vertical transmission of gut microbiota. The overall GM composition could stably persist up to 11 generations with inbred mice (Moeller et al., 2018). The activity of *Lb. rhamnosus* indicates its robustness to change microbiota in the next generation by colonizing it during pregnancy, but it remains unknown if this functioning could be transferred to other lactobacillus or the* bona fide* route where transmission happens.

*Lb. rhamnosus* is a species of lactic acid bacteria with typical probiotic properties. Among them, its role in controlling the growth of pathogens is increasingly appreciated (Kohler et al., 2012). One of the most prominent capacities was assigned to the inactivation of *E. coli*, whereas LGR-1 was shown to ameliorate *E. coli*-induced inflammation and cell damage during inflammasome activation (Wu et al., 2015). A similar potency could also be applied to inflammatory responses in bovine endometrial epithelial cells (Liu et al., 2016), activation of urinary bladder cells (Karlsson et al., 2012), etc. These results are consistent with the current finding that *Lb. rhamnosus* improves spatial memory damaged or aggravated by *E. coli*, at a very early stage of microbiota development. The *in vitro* study clarified the direct interaction of these two microbes. Still, although O157 infection, just like *Citrobacter rodentium* (Gareau et al., 2011), damaged spatial memory, it could not be excluded that other bacteria, susceptible to probiotic intervention, also contributed to the consequent CNS functioning.

In conclusion, *Lacticaseibacillus rhamnosus* prevents memory dysfunction caused by lead exposure in female rats. This prophylactic effect was likely to be achieved by finetuning gut microbiota characterized by the deceased abundance of *E. coli*. This study offers an example of using a single probiotic bacterium to interfere with neuropathy in early life. This study has some limitations exemplified by gender preference, discrepancy of correlation factored by sampling time, etc., which might request further interrogation. Considering that nutritional prevention has the advantage of avoiding the initial hazard, this microbe-based strategy is promising in ameliorating cognitive impairment with environmental cues.

## Data availability statement

The original contributions presented in the study are publicly available. This data can be found here: https://www.ncbi.nlm.nih.gov/bioproject/PRJNA906312.

## Ethics statement

The animal study was reviewed and approved by the Institutional Animal Care and Use Committee of Hefei University of Technology.

## Author contributions

YX and YN conceived and designed the study. JZ conducted the majority of research and summarized data. ZH, LL, and HL performed the *in vitro* experiments. TW, XZ, YW, and DZ assisted in animal experiments. YX wrote the article that was reviewed by all authors. All authors contributed to the article and approved the submitted version.

## Supplementary material

Supplementary Figure S1 Total distance traveled during water maze trial (*n* = 5–7). Statistical analysis was performed using one-way ANOVA or unpaired *t-*test. All data are expressed as mean ± SEM. ctrl, untreated rats; Pb, lead-exposed rats during lactation. Gestation, lactation, and postweaning represent the treating period of LGR-1, respectively.

Supplementary Figure S2 Microbiome analysis upon probiotic intervention. **(A)** α-diversity of gut microbiota was shown upon various treatments. **(B–D)** Relative abundance of Proteobacteria **(B)**, Firmicutes **(C)**, and Firmicutes/Bacteroidota ratio **(D)** across treatments, as revealed by 16S rRNA sequencing. **(E)** The relative abundance of lactobacillus across groups, as revealed by 16S rRNA sequencing (*n* = 5–6). Statistical analysis was performed using one-way ANOVA or unpaired *t-*test. All data are expressed as mean ± SEM. **P* < 0.05; ***P* < 0.01; ****P* < 0.001. ctrl, untreated rats; Pb, lead-exposed rats during lactation; G, *Lacticaseibacillus rhamnosus* GR-1; P, prevention mode indicating gestational supplementation of probiotic; R, repair mode indicating postweaning supplementation of probiotic.

Supplementary Figure S3 *In vitro* inhibition of *Lb. rhamnosus* towards *E. coli* O157. **(A–C)** The influence of incubation time of LGR-1 on the viability of O157. The co-culture was incubated for 6 h **(A)**, 12 h **(B)**, and 18 h **(C)**, respectively before being subjected to plate counting of *E. coli*. **(D–F)** The influence of pH on the inhibitory effect. The MRS-LB medium was adjusted to pH 6 (D), pH 7 **(E)**, and pH 8 **(F)**. Panel **(G)** represents the direct comparison under varying pH conditions, in the context of 100 μl LGR-1 and 18 h incubation. Statistical analysis was performed using one-way ANOVA or unpaired *t*-test. All data are expressed as mean ± SEM. **P* < 0.05; ***P* < 0.01; ****P* < 0.001, *****P* < 0.0001. LGR-1, *Lacticaseibacillus rhamnosus* GR-1.

Supplementary Figure S4 Membrane penetrance influenced by *Lb. rhamnosus* on *E. coli* O157. Panel **(A)** represents PI staining of *E. coli* to indicate membrane penetrance. Panel **(B)** represents the fluorescence intensity calculated from five respective images for each group using ImageJ software. The number refers to the volume of LGR-1 culture added to the system. Statistical analysis was performed using one-way ANOVA. All data are expressed as mean ± SEM. **P* < 0.05; ***P* < 0.01; ****P* < 0.001.
