# Supplementary figures and images for: Probiotic has prophylactic effect on spatial memory deficits by modulating gut microbiota characterized by the inhibitory growth of Escherichia coli
Source: Front Integr Neurosci. 2023 Feb 21;17:1090294. doi: 10.3389/fnint.2023.1090294 (PMC9990170; doi:10.3389/fnint.2023.1090294)

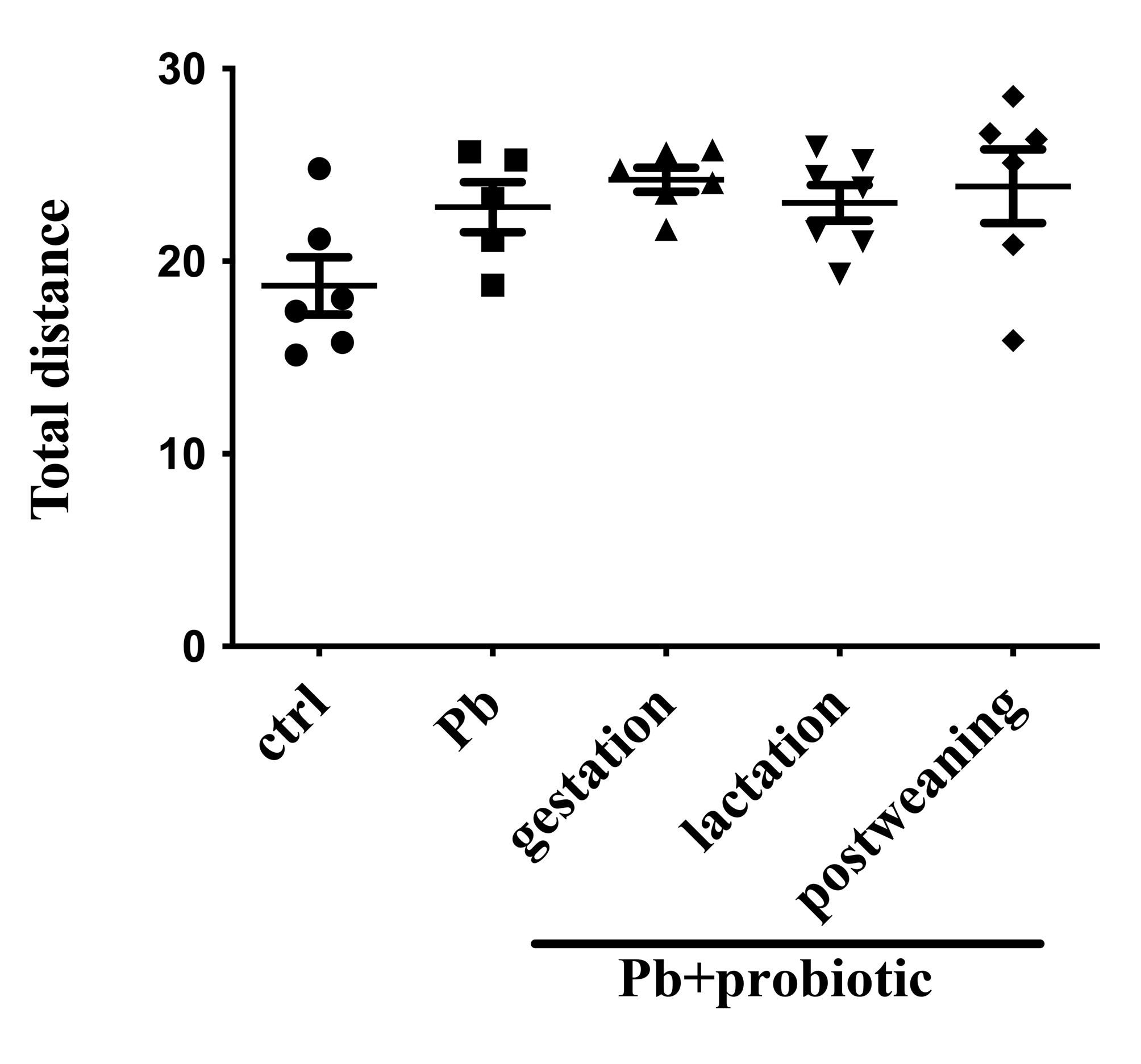

Supplement: Supplementary Figure S1 — Total distance traveled during water maze trial (n = 5–7). Statistical analysis was performed using one-way ANOVA or unpaired t-test. All data are expressed as mean ± SEM. ctrl, untreated rats; Pb, lead-exposed rats during lactation. Gestation, lactation, and postweaning represent the treating period of LGR-1, respectively. [file Image_1.TIF]

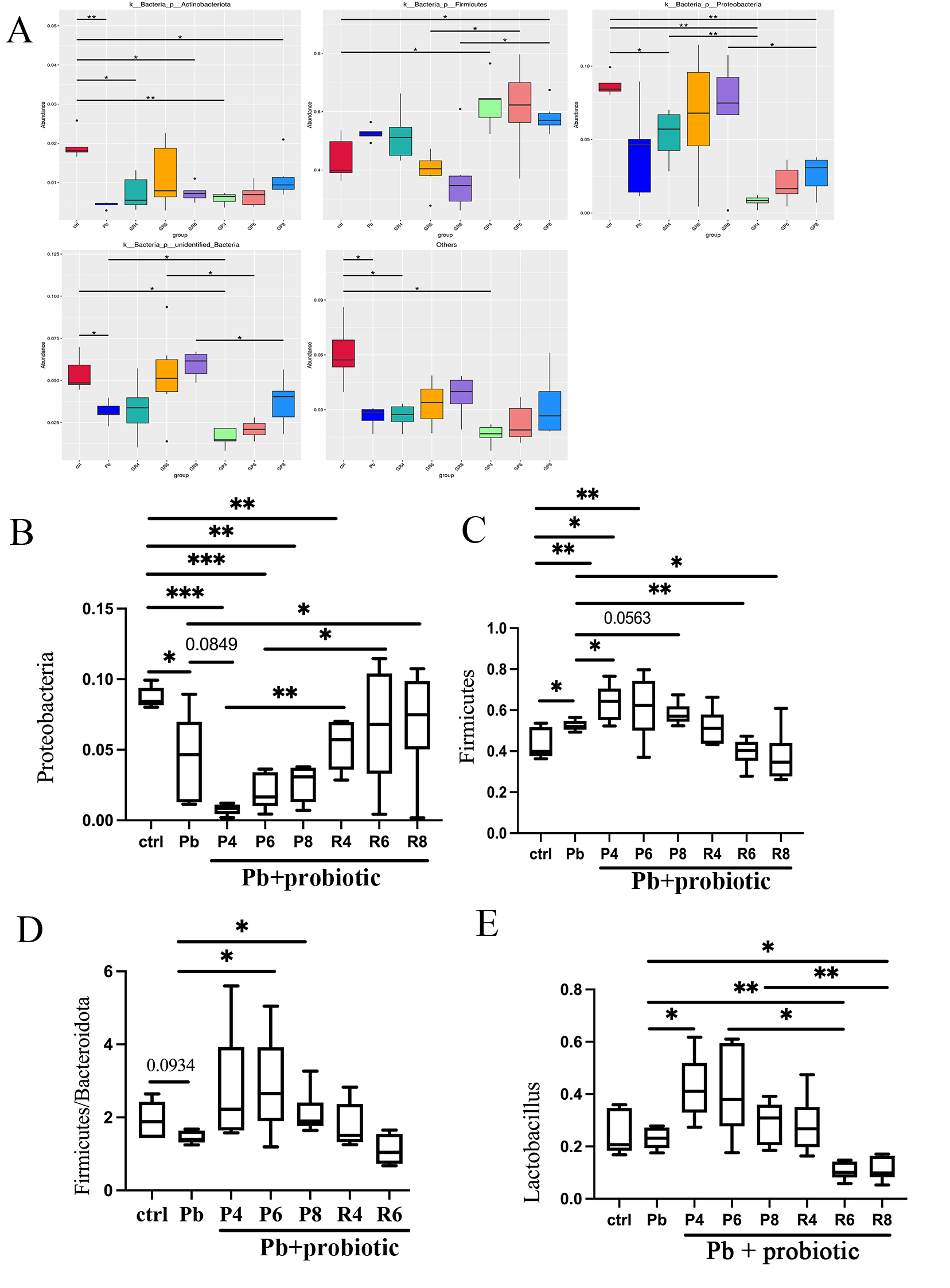

Supplement: Supplementary Figure S2 — Microbiome analysis upon probiotic intervention. (A) α-diversity of gut microbiota was shown upon various treatments. (B–D) Relative abundance of Proteobacteria (B), Firmicutes (C), and Firmicutes/Bacteroidota ratio (D) across treatments, as revealed by 16S rRNA sequencing. (E) The relative abundance of lactobacillus across groups, as revealed by 16S rRNA sequencing (n = 5–6). Statistical analysis was performed using one-way ANOVA or unpaired t-test. All data are expressed as mean ± SEM. *P < 0.05; **P < 0.01; ***P < 0.001. ctrl, untreated rats; Pb, lead-exposed rats during lactation; G, Lacticaseibacillus rhamnosus GR-1; P, prevention mode indicating gestational supplementation of probiotic; R, repair mode indicating postweaning supplementation of probiotic. [file Image_2.TIF]

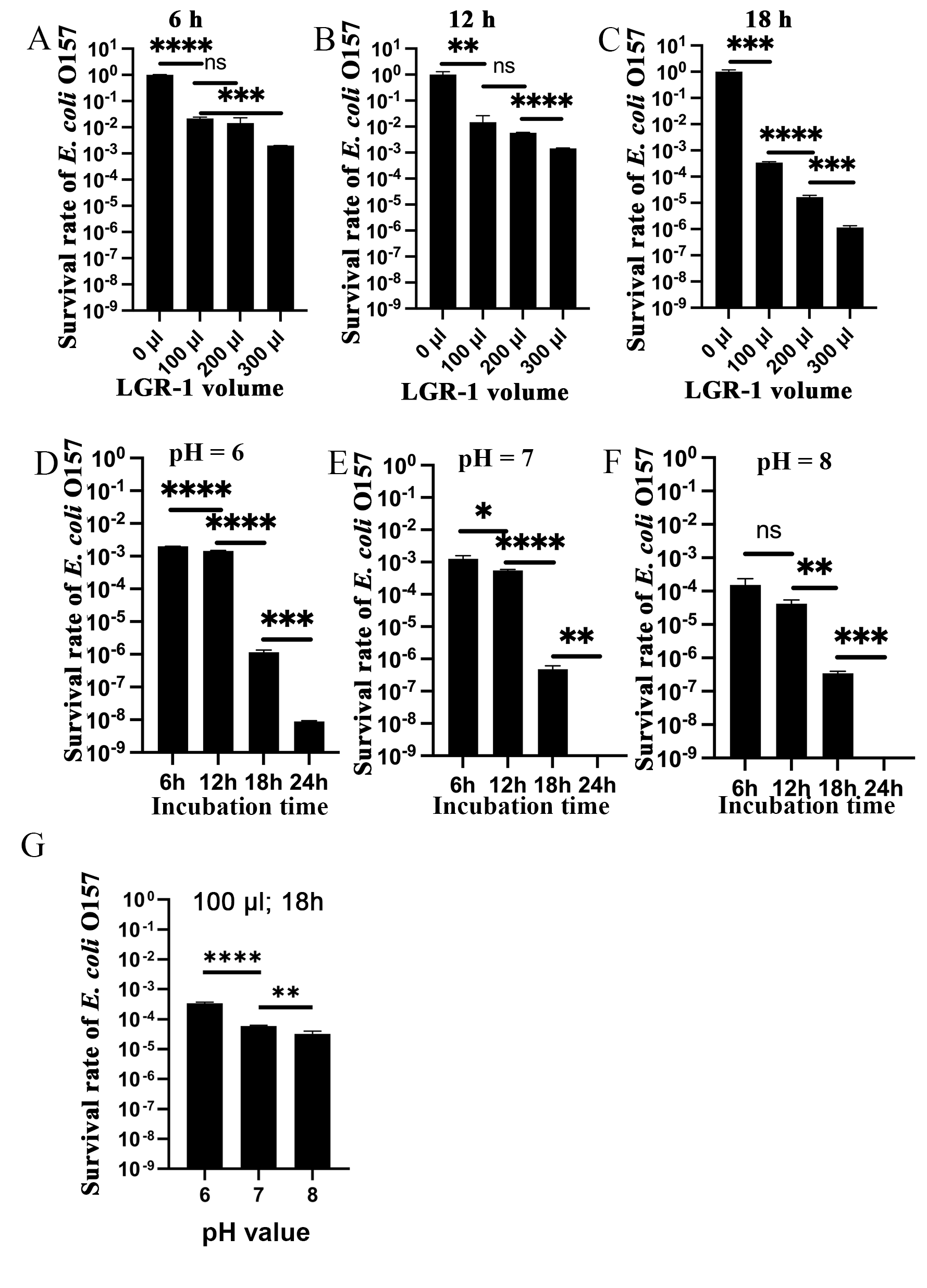

Supplement: Supplementary Figure S3 — In vitro inhibition of Lb. rhamnosus towards E. coli O157. (A–C) The influence of incubation time of LGR-1 on the viability of O157. The co-culture was incubated for 6 h (A), 12 h (B), and 18 h (C), respectively before being subjected to plate counting of E. coli. (D–F) The influence of pH on the inhibitory effect. The MRS-LB medium was adjusted to pH 6 (D), pH 7 (E), and pH 8 (F). Panel (G) represents the direct comparison under varying pH conditions, in the context of 100 μl LGR-1 and 18 h incubation. Statistical analysis was performed using one-way ANOVA or unpaired t-test. All data are expressed as mean ± SEM. *P < 0.05; **P < 0.01; ***P < 0.001, ****P < 0.0001. LGR-1, Lacticaseibacillus rhamnosus GR-1. [file Image_3.TIF]

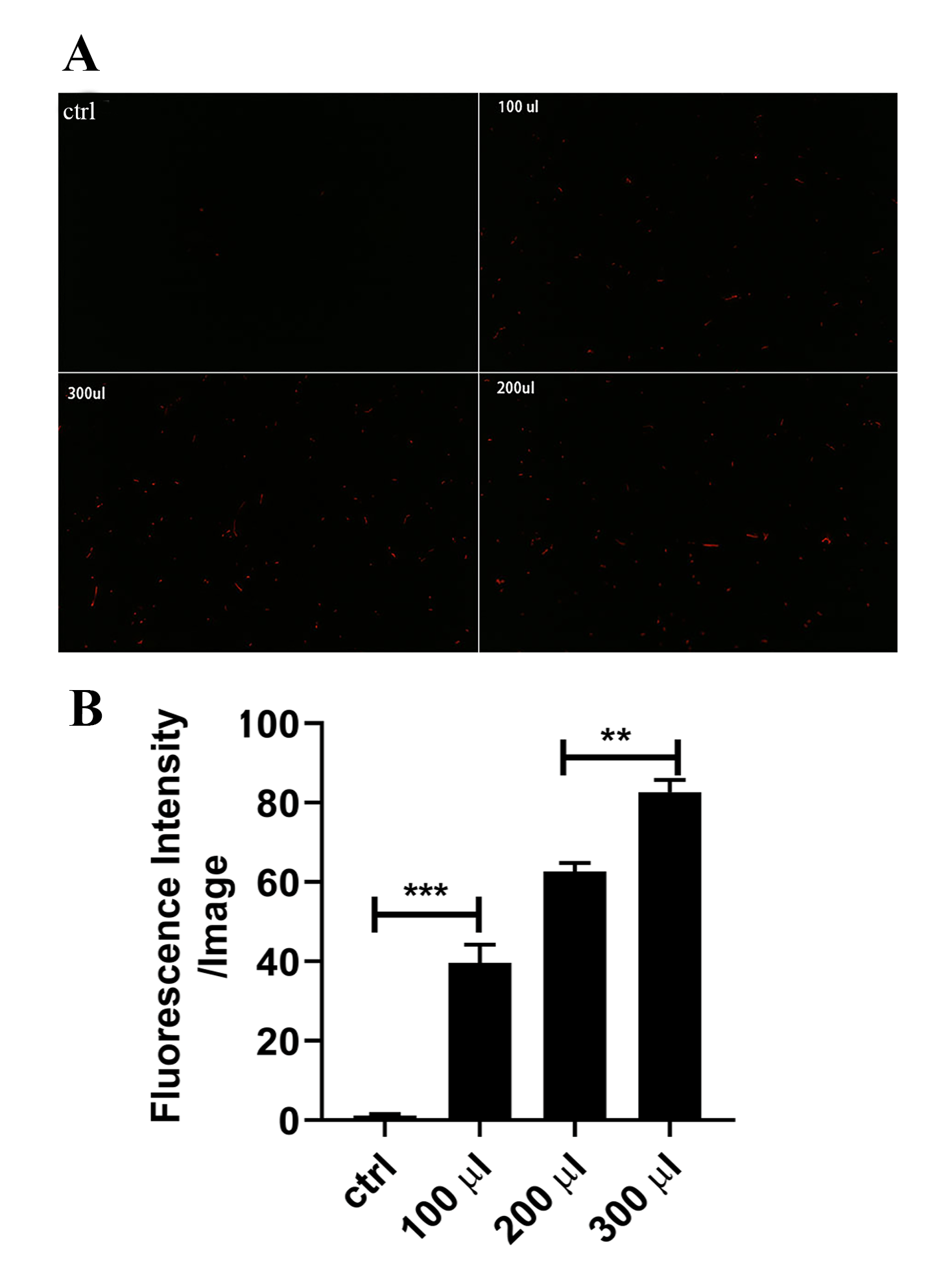

Supplement: Supplementary Figure S4 — Membrane penetrance influenced by Lb. rhamnosus on E. coli O157. Panel (A) represents PI staining of E. coli to indicate membrane penetrance. Panel (B) represents the fluorescence intensity calculated from five respective images for each group using ImageJ software. The number refers to the volume of LGR-1 culture added to the system. Statistical analysis was performed using one-way ANOVA. All data are expressed as mean ± SEM. *P < 0.05; **P < 0.01; ***P < 0.001. [file Image_4.TIF]
